# CXCL5 overexpression predicts a poor prognosis in pancreatic ductal adenocarcinoma and is correlated with immune cell infiltration

**DOI:** 10.7150/jca.40517

**Published:** 2020-02-10

**Authors:** Ronghua Zhang, Qiaofei Liu, Junya Peng, Mengyi Wang, Tong Li, Jingkai Liu, Ming Cui, Xiang Zhang, Xiang Gao, Quan Liao, Yupei Zhao

**Affiliations:** 1 Department of General Surgery, Peking Union Medical College Hospital, Peking Union Medical College & Chinese Academy of Medical Sciences, Beijing 100730, China; 2 Department of Medical Research Center, Peking Union Medical College Hospital, Peking Union Medical College & Chinese Academy of Medical Sciences, Beijing 100730, China

**Keywords:** CXCL5, pancreatic ductal adenocarcinoma, prognosis, immune cell infiltration

## Abstract

**Background**: C-X-C motif chemokine 5 (CXCL5) is an important attractant for immune cell accumulation in tumor tissues. Recent evidence has shown that CXCL5 could promote carcinogenesis and cancer progression in a variety of cancer types. However, the relationships between CXCL5, immune cell infiltration and pancreatic ductal adenocarcinoma (PDAC) remain largely unknown. This study aimed to explore the role and regulative mechanism of CXCL5 in PDAC carcinogenesis.

**Materials and Methods**: The expression of CXCL5 in PDAC was analyzed based on online databases and tissue microarray staining, and Western blotting of CXCL5 in PDAC cell lines and patient samples. The correlation between CXCL5 expression and clinicopathological features, prognosis and immune cell infiltration in tumor tissues was analyzed.

**Results**: High expression of CXCL5 was observed both in PDAC tumor tissue and PDAC cell lines, compared to normal pancreas tissues and normal ductal epithelium cells. High CXCL5 expression in tumor tissues was positively correlated with an advanced T stage (p=0.036), a positive tumor lymph node metastasis (p=0.014), a poor differentiation status (p=0.003) and a poor prognosis (p=0.001). Combination of CA242 and CXCL5 expression (p<0.0001) served as a better prognostic factor than CA242 alone (p=0.006). In addition, PDAC patients with high CXCL5 expression had more intratumoral M2 polarized macrophages (p=0.0248), neutrophils (p=0.0068) and IgG^+^ plasma cells (p=0.0133) than patients with low CXCL5 expression.

**Conclusions**: The expression of CXCL5 is elevated in pancreatic cancer cells. High CXCL5 expression is positively correlated with poor survival and the increased infiltration of several types of immune suppressive cells. Thus, CXCL5 could be a promising therapeutic target for PDAC immunotherapy.

## Introduction

Pancreatic cancer is one of the leading causes of tumor deaths, with rising incidence and mortality^1^, ^2^. Pancreatic ductal adenocarcinoma (PDAC) accounts for 90% of pancreatic cancer^3^. Although the survival of PDAC patients has improved due to advances in surgical management, the overall 5-year survival rate of patients after radical resection is only 20%-40%, due to the high recurrence rate^4^. PDAC is featured by a unique tumor immune microenvironment (TIME), characterized by pronounced desmoplasia and immune cell infiltration^5^. The TIME is composed of cancer cells and stromal cells, including tumor-associated neutrophils (TANs), tumor-associated macrophages (TAMs), regulatory T cells (Tregs), dendritic cells and tumor-associated B-cells^6^, ^7^. Interactions between stromal cells and cancer cells plays vital roles in the processes of PDAC carcinogenesis^8^. Thus, a better understanding of the pathogenesis of PDAC and the functions of the TIME is urgently needed.

Comprehensive network between chemokines and related receptors was reported to shape TIME thus facilitating cancer progression^9^. C-X-C motif chemokine 5 (CXCL5), is a member of the C-X-C chemokine family, that acts as an important attractant for granulocytic immune cells by binding to its receptor C-X-C Chemokine Receptor Type 2 (CXCR2)^10^. CXCL5 overexpression has been observed in several malignancies, including osteosarcoma, glioma, and lung, bladder, liver, prostate and colorectal cancers^10^-^16^, demonstrating its roles in tumor carcinogenesis. Furthermore, CXCL5-CXCR2-dominated cross-talk between cancer cells and macrophages or neutrophils could promote tumor metastases in gastric, hepatocellular and prostate cancers^12^, ^17^, ^18^. However, the roles of CXCL5 in PDAC and the relationships between CXCL5 and the TIME are largely unknown.

In this study, we explored the expression of CXCL5 in PDAC tissues and its relationship with the clinicopathological features and prognosis of PDAC patients. We further investigated the correlation between CXCL5 abundance and the infiltration of immune cells in the TIME.

## Material and Methods

### Database analysis

The expression levels of CXCL5 in various types of cancers were analyzed with the online database Gene Expression Profiling Interactive Analysis (GEPIA) (http://gepia.cancer-pku.cn/index.html), which is a web server for cancer and normal gene expression profiling^19^. We compared CXCL5 mRNA levels in cancer vs. normal patient datasets using the Oncomine database, which is a cancer microarray database (https://www.oncomine.org/resource/login.html) 20. The differentially expressed genes (CXCL5) between 69 pancreatic tumors and 61 adjacent nontumor tissues were also analyzed using microarray data obtained from the Gene Expression Omnibus (GEO) database (GSE62452). We also used the online biomarker validation tool SurvExpress^21^ to evaluate the relationship between CXCL5 expression and cumulative survival and risks in The Cancer Genome Atlas (TCGA) pancreatic carcinoma dataset. The CXCL5 expression levels of PDAC patients in the dataset were divided into “Low Risk” and “High Risk” groups according to the prognostic index (http://bioinformatica.mty.itesm.mx:8080/Biomatec/SurvivaX.jsp). The prognostic significance of CXCL5 mRNA expression in pancreatic cancer patients was evaluated by Kaplan-Meier plotter (http://kmplot.com), a widely used online database.

### Cell culture

Six human pancreatic cancer cell lines (BxPC-3, Mia PaCa-2, SW1990, PANC-1, AsPC-1 and CFPAC-1) and the normal pancreatic ductal epithelial cell line HPDE6-C7, were cultured in a humidified incubator with 5% CO_2_ at 37 °C in Dulbecco's modified Eagle's medium (DMEM, HyClone) or RPMI-1640 medium containing 10% fetal bovine serum (FBS, Gibco).

### PDAC sample collection and tissue microarray construction

Ninety patients with PDAC treated in Peking Union Medical College Hospital were examined. The inclusion criteria were as follows: (1) all the patients underwent R0 pancreaticoduodenectomy (1 mm without cancer cells); (2) no neoadjuvant treatment was performed; (3) clinicopathological information and a follow-up visit were available; (4) all the tumor tissues were pathologically confirmed as PDAC; and (5) all the patients received at least three courses of gemcitabine-based treatment. Eight clinicopathological items, including age, gender, T stage and N stage (according to the 8th edition of the TNM staging system), differentiation grade, perineural invasion, CA19-9, and CA242, were analyzed. This study was approved by the Ethics Committee of Peking Union Medical College Hospital (registration number is NCT02654288). All the patients signed the informed consent form. The mean age of the patients was 60 years and the male to female ratio was 57:33. While 60 of 90 patients died, the remaining 30 patients were still alive, between 6 and 92 months after resection. Tissue microarrays were constructed using formalin-fixed paraffin-embedded blocks. In addition, seven paired samples, including tumor tissue and corresponding nontumor normal tissues from patients with PDAC were obtained for further study.

### Immunohistochemistry (IHC) assays and evaluation

Immunohistochemical staining was routinely performed according to a standard protocol. Tissue samples were stained with hematoxylin and eosin to confirm the histological type of PDAC. CXCL5 antibody (1:100, ab9802, Abcam) was used. For the immunohistochemical scoring of CXCL5, the staining intensity was scored as 0 (negative), 1 (weak), 2 (moderate) and 3 (strong) and the proportions of cancer cells were classified as 0 (0-5%), 1 (6%-25%), 2 (26%-50%), 3 (51% to 75%) and 4 (76% to 100%). The multiplication for intensity and proportion was used to evaluate the expression level of CXCL5. According to the largest Youden's index for each variable within the receiver operating characteristic (ROC) curve, the median score of CXCL5 was 4. A score less than 4 was defined as low expression, while a score greater than or equal to 4 was defined as high expression. Immune cells were stained with different immune cell markers, as follows: CD68 (IR613, DAKO) for macrophages, CD163 (Ab182422, Abcam) for M2-polarized macrophages, CD15 (IR062, DAKO) for neutrophils, S-100 (IR504, DAKO) for dendritic cells, CD3 (RTU-CD3-PSI, Leica) for T lymphocytes, CD4 (PA0427, Leica) for helper T lymphocytes, CD8 (IR623, DAKO) for cytotoxic T lymphocytes, FOXP3 (Ab20034, Abcam) for Tregs, CD56 (RTU-CD56-1B6) for natural killer cells, CD19 (IR656, DAKO) for B lymphocytes, IgG (IR512, DAKO) for plasma cells and IgG4 (GI-0910, GENEMED) for suppressive plasma cells. Six areas with abundant immune cell infiltration were selected at a low-power field, the number of positive cells in each field was counted, and the average count/ high-power field (HFP) (400× magnification) was calculated^22^.

### Western blot analysis

Western blotting was performed according to a standard protocol. Total cell or tissue lysates were extracted using 2% SDS lysis buffer (Applygen, Beijing), and 30 μg of total proteins were separated on 12% (v/v) SDS-PAGE gels. After electrophoresis, the proteins were transferred onto nitrocellulose membranes (Millipore, Ireland) and the membrane was incubated with the anti-CXCL5 antibody (1:500, ab9802, Abcam) and anti-GAPDH antibody (1:1000, H-12; Santa Cruz Biotechnology) at 4 ℃ overnight. After washing three times, the membranes were incubated with horseradish peroxidase (HRP)-conjugated secondary antibodies (1:2000, Zsbio, Beijing) for 1 h at room temperature. Finally, the membranes were visualized using an ECL Kit (Applygen, Beijing).

### Statistical analysis

All data were analyzed by IBM SPSS Statistics software version 21.0 and GraphPad Prism software version 5.0. The scores of CXCL5 staining in tumor and nontumor tissues in the GSE62452 database and the correlation analysis between CXCL5 and immune cell infiltration in tumor tissue were compared using the Mann-Whitney U test. Overall survival (OS) was analyzed using the Kaplan-Meier method, and the differences in OS were measured by the log-rank test. The Fisher exact test and the Pearson chi-square test were used to analyze association among variables. A multivariable analysis was performed using the Cox proportional hazards regression method. Spearman correlation analysis was used to analyze the correlation between the expression of genes. A two-tailed p-value < 0.05 was considered significant.

## Results

### Increased expression of CXCL5 in pancreatic cancer

**Figure 1:**
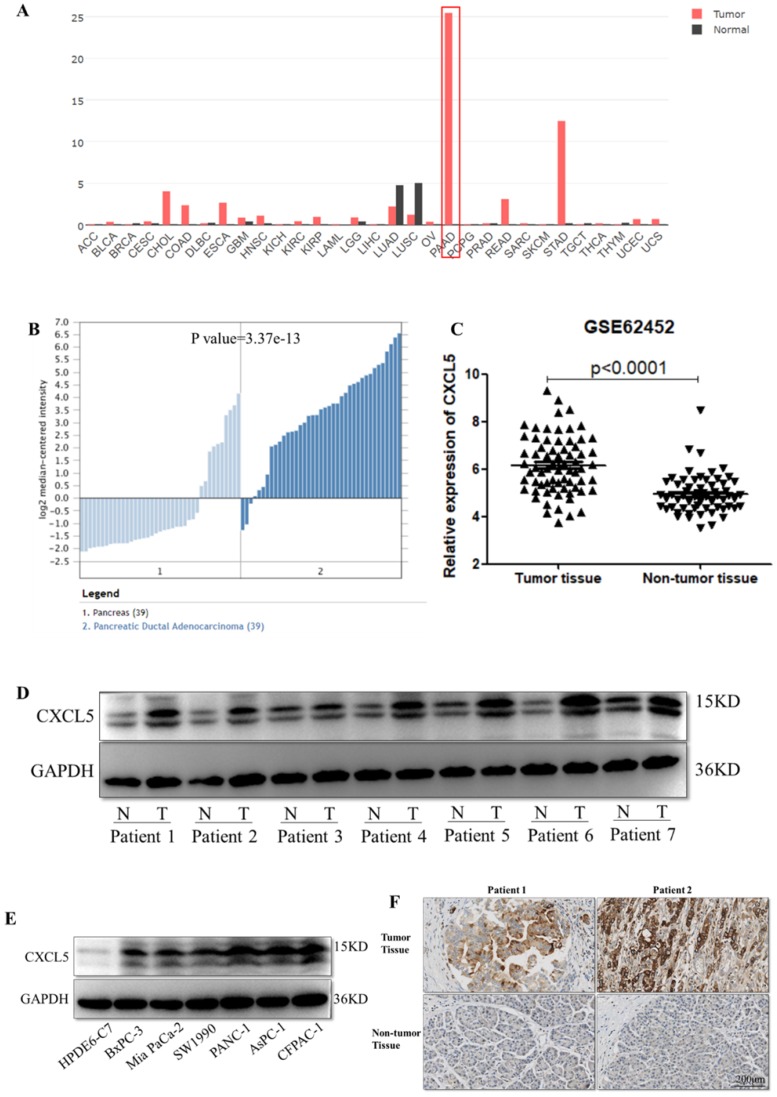
** Expression of CXCL5 in PDAC tissues.** (A) Expression of CXCL5 in tumor and normal tissues in multiple cancer types using the GEPIA database based on TCGA and GTEx data. (B) The differences of CXCL5 expression in PDAC and normal pancreas tissues using the Oncomine and GEO databases. (C) Expression of CXCL5 in pancreatic tumor tissues and adjacent nontumor tissues in the GSE62452 database. (D) Expression of CXCL5 in paired tumor tissues and adjacent nontumor tissues from seven PDAC patients (E) Expression of CXCL5 in one normal pancreatic ductal epithelial cell line and six pancreatic cancer cell lines detected by Western blot analysis. (F) Representative images of CXCL5 in tumor tissues and the adjacent nontumor tissues.

To determine the differences of CXCL5 expression in tumor and normal tissues, the CXCL5 expression levels in multiple cancer types were analyzed using the GEPIA database based on TCGA and Genotype-Tissue Expression (GTEx) data. The data revealed types of tumors express higher CXCL5 compared to related normal tissues, including pancreatic adenocarcinoma (PAAD), in cholangiocarcinoma (CHOL), colon adenocarcinoma (COAD), esophageal carcinoma (ESCA), rectum adenocarcinoma (READ) and stomach adenocarcinoma (STAD) tissues. Among these tumors, differential gene expression of CXCL5 is highest in PAAD (Fig. 1A). We further investigated the differences of CXCL5 mRNA expression in PDAC using the Oncomine and GEO databases. As shown in Fig. 1B, CXCL5 expression in tumor tissue was elevated compared to that in normal pancreatic tissue. Analysis of the gene expression profile of 69 pancreatic tumor tissues and 61 adjacent nontumor tissues in the GSE62452 database, revealed that the expression of CXCL5 was significantly higher in pancreatic cancer tissues than in adjacent nontumor tissues (p<0.0001, Mann-Whitney *U* test, Fig. 1C). To evaluate the protein abundance of CXCL5 expression in cancer tissues and nontumor tissues, the expression of CXCL5 was detected by Western blotting in paired PDAC tissue samples and cell lines. Higher CXCL5 protein abundance was observed in seven PDAC tumor tissues and six cell lines compared to adjacent non-tumor tissues or normal pancreatic ductal epithelial cell line HPDE6-C7 (Fig. 1D, E). We also applied CXCL5 staining in paired PDAC patient tissues and observed higher expression of CXCL5 in neoplastic tissues (Fig. 1F). These data suggested that CXCL5 expression was increased in PDAC.

### Correlation between CXCL5 expression and clinicopathological features

**Figure 2:**
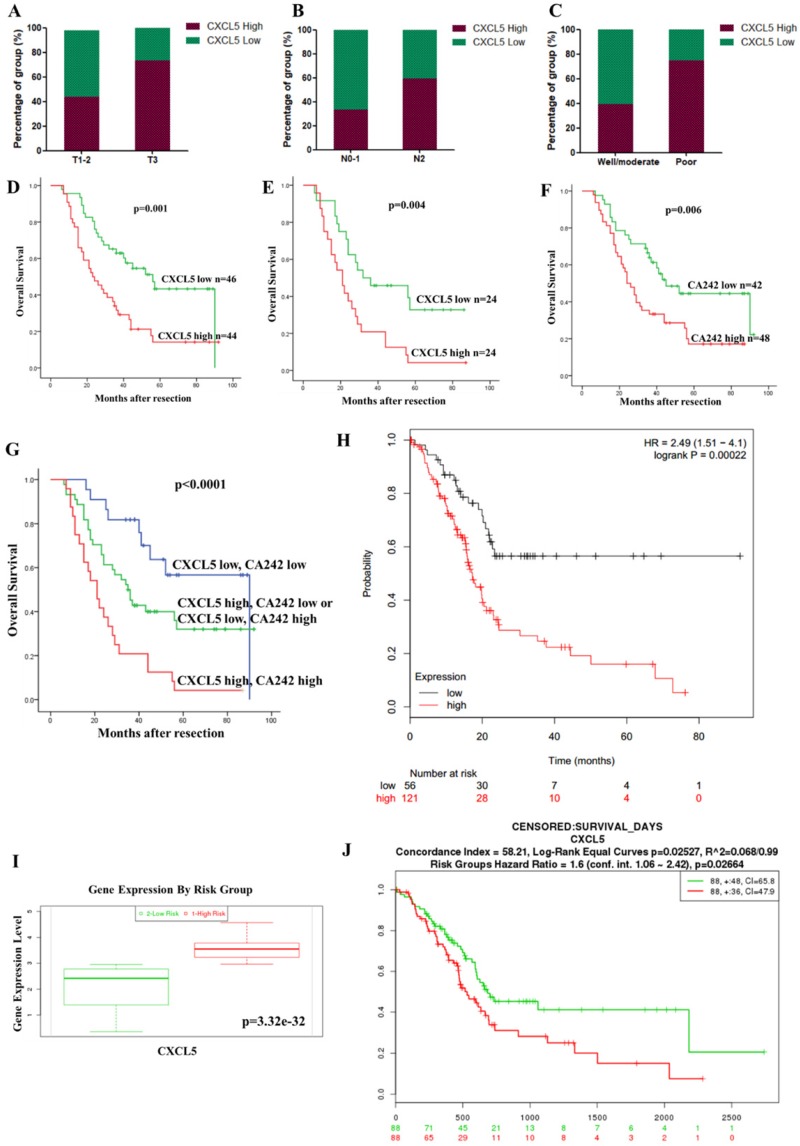
** Correlation between CXCL5 expression and clinicopathological features and prognosis.** (A-C) Expression of CXCL5 in tumor tissues with different T stages, N stages and differentiation statuses. (D) The influence of tumoral CXCL5 expression on OS (p=0.001, log-rank test). (E) The multivariate Cox regression analysis showed that CXCL5 expression was an independent factor in the high CA242 subgroup (HR=2.047, p=0.004). (F) PDAC patients with high CA242 levels had worse OS than those with low CA242 levels (p=0.006). (G) The combination of CXCL5 expression and CA242 was a good prognostic factor (p<0.0001). (H) The results from SurvExpress, showed that high CXCL5 expression was associated with poor OS in PDAC patients (p=0.00022). (I) Comparison of CXCL5 expression between patients in the “High Risk” group and those in the “Low Risk” group through the SurvExpress program (p=3.32e^-32^). (J) The prognostic value of CXCL5 mRNA expression in the Kaplan-Meier plotter dataset (HR=1.6, p=0.02664).

We investigated the relationship between CXCL5 expression and clinicopathological features. High expression of CXCL5 in tumor tissues was positively associated with an advanced T stage (p=0.036), positive lymph node metastasis (p=0.014) and a poor differentiation status (p=0.003) in 90 PDAC patients (Table 1, Fig. 2A, 2B and 2C). This result suggests CXCL5 expression is clinically relevant.

### CXCL5 overexpression in tumor tissues was associated with a poor prognosis of PDAC patients

We further investigated the correlation between CXCL5 expression and the prognosis of PDAC patients. The univariate analysis showed that high CXCL5 expression in tumor tissues (p=0.001, Fig. 2D and Table 2), the T stage (p=0.004), the N stage (p<0.001), the differentiation status (p=0.001), and high level of CA19-9 (p=0.001) and CA242 (p=0.008) were significantly associated with poor OS (Table 2). The multivariate Cox regression analysis indicated that the T stage (p=0.007), the N stage (p=0.012) the differentiation status (p=0.004), and high level of CA19-9 (p=0.030) were independent prognostic factors (Table 2) and CXCL5 expression was identified as an independent factor in the high CA242 subgroup (HR=2.047, p=0.004, Fig. 2E). These results indicated that the combination of CA242 and CXCL5 expression (p<0.0001, Fig. 2G) was a better prognostic factor than CA242 alone (p=0.006, Fig. 2F). The results from the SurvExpress also showed that high CXCL5 expression was associated with poor OS in PDAC patients (p=0.00022, Fig. 2H). CXCL5 expression was significantly elevated in the “High Risk” group (p=3.32e^-32^, Fig. 2I). In the Kaplan-Meier plotter databases, high CXCL5 mRNA expression was found to be correlated with significantly shorter OS among 176 PDAC patients (p=0.02664, Fig. 2J). Overall, CXCL5 overexpression may be a biomarker that indicates poor survival for PDAC patients, especially in the high CA242 group.

### CXCL5 expression related to infiltrated immune cells in PDAC

**Figure 3:**
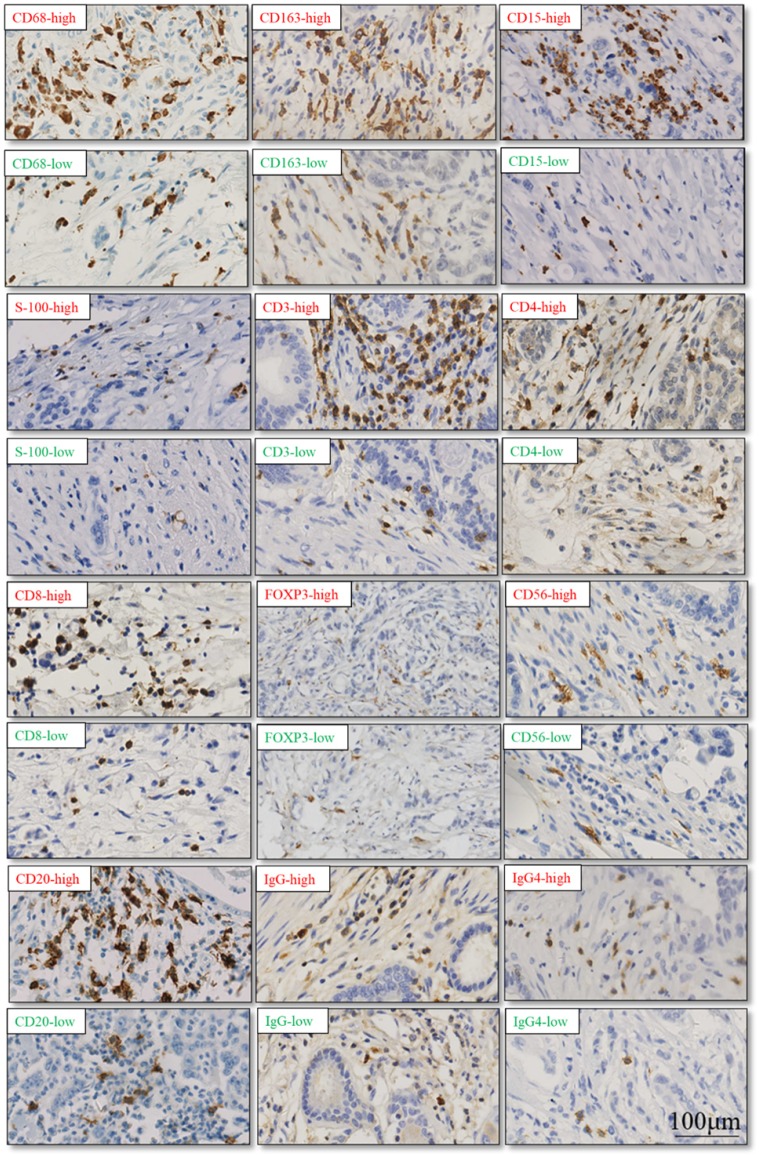
** Representative cases of immunohistochemical staining of 12 immune cell populations.** (red: high level of immune cell infiltration; green: low level of immune cell infiltration)

**Figure 4:**
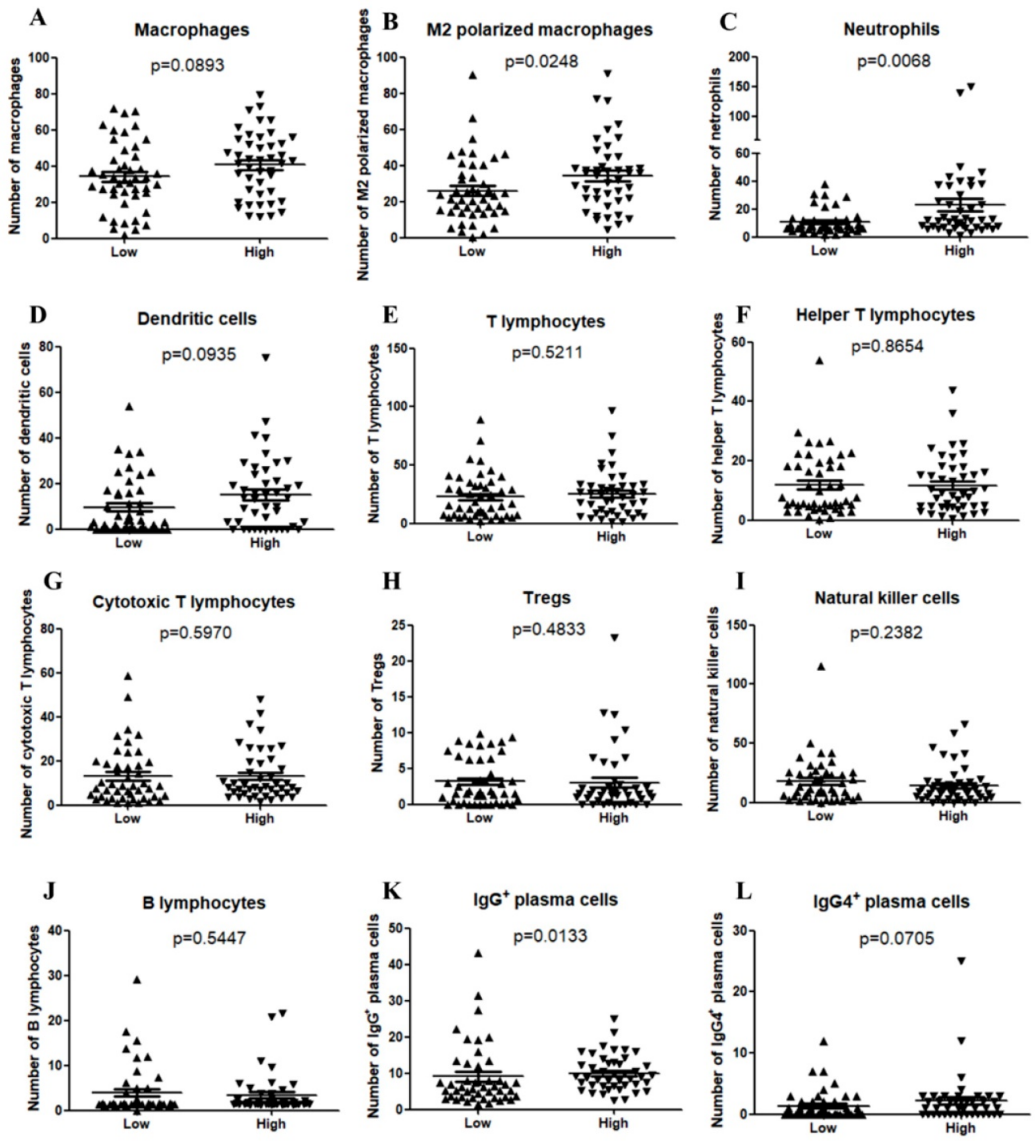
** Correlation between CXCL5 expression and immune cell infiltration in PDAC samples.** (A) Macrophages (p=0.0893); (B) M2 polarized macrophages (p=0.0248); (C) Neutrophils (p=0.0068); (D) Dendritic cells (p=0.0935); (E) T lymphocytes (p=0.5211); (F) Helper T lymphocytes (p=0.8654); (G) Cytotoxic T lymphocytes (p=0.5970); (H) Tregs (p=0.4833); (I) Natural killer cells (p=0.2382); (J) B lymphocytes (p=0.5447); (K) IgG^+^ plasma cells (p=0.0133); (L) IgG4^+^ plasma cells (p=0.0705).

**Figure 5:**
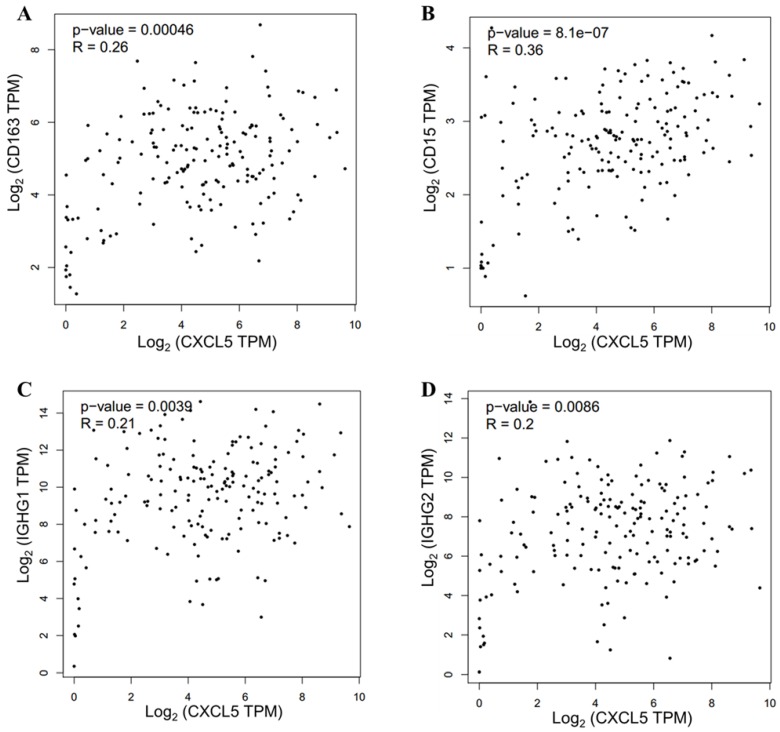
** Validation of correlation between CXCL5 expression and infiltration of M2 polarized macrophages, neutrophils and IgG^+^ plasma cells in pancreatic cancer from data of TCGA and GTEx.** (A) Correlation analysis between CXCL5 and CD163 (R=0.26, p=0.00046, Spearman correlation analysis); (B) Correlation analysis between CXCL5 and CD15 (R=0.46, p=8.1× e^-7^, Spearman correlation analysis). (C) Correlation analysis between CXCL5 and IGHG1 (R=0.21, p=0.0039, Spearman correlation analysis). (D) Correlation analysis between CXCL5 and IGHG2 (R=0.2, p=0.0086, Spearman correlation analysis). Data was obtained from the GEPIA (http://gepia.cancer-pku.cn/).

The TIME has been reported to play vital roles in the biological behaviors of PDAC^8^. We further investigated the relationship between CXCL5 expression and infiltrated immune cells in PDAC. We performed serial immunohistochemical staining of 12 immune populations in the PDAC tissue microarray (Fig.3). Infiltration of immune suppressive cells, such as M2 polarized macrophages (p=0.0248, Fig. 4B), neutrophils (p=0.0068, Fig. 4C) and IgG^+^ plasma cells (p=0.0133, Fig. 4K), was enriched in patients with high CXCL5 expression. Other types of immune cells, such as T lymphocytes, dendrite cells, natural killer cells and B lymphocytes, which infiltration were not related to CXCL5 abundance. To validate our findings, we analyzed the correlation between CXCL5 expression and infiltration of these immune cells in pancreatic cancer from data of TCGA and GTEx. The infiltration of M2 polarized macrophages (R=0.26, p=0.00046, Fig. 5A) and neutrophils (R=0.36, p=8.1× e^-7^, Fig. 5B) was correlated with the expression of CXCL5 in pancreatic cancer tissues. Due to the lack of corresponding gene description of IgG in the TCGA databases, we analyzed the expression of immunoglobulin heavy constant gamma 1 (IGHG1) and IGHG2, which make up 90% of the four IgG subclasses (IgG1, IgG2, IgG3 and IgG4)^23^, to represent the expression of IgG. We found that the expression of IGHG1(R=0.21, p=0.0039, Fig. 5C) and IGHG2 (R=0.2, p=0.0086, Fig. 5D) was both correlated with the expression of CXCL5. These data suggested that high CXCL5 expression could probably link immune suppression phenotype to contribute to the poor prognosis of PDAC patients, but further studies are needed to determine whether CXCL5 could mediate the phenotypes and functions of immune cells.

## Discussion

PDAC is characterized by immunosuppressive microenvironment that constitutes the main obstacle to effective PDAC immunotherapy. An immunosuppressive TIME plays vital roles in cancer progression, and immunotherapy is one of the emerging therapeutic options for PDAC^24^. Therefore, the identification of novel biomarkers for the detection of PDAC and targets for immunotherapy is urgently needed to improve the prognosis of patients. In this study, we found CXCL5 expression was dysregulated in PDAC tumor tissues and its abundance correlated with PDAC outcome. Notably, CXCL5 expression was observed related to infiltrated suppressive immune cells, such as M2 polarized macrophages, neutrophils, IgG^+^ plasma cells. This study suggests CXCL5 plays a key role in TIME shaping and can be served as potential novel prognosis marker.

Roles of CXCL5 are different in tumor progression due to types of responsive cells in different tumors. CXCL5 can promote tumor progression in types of cancers by promoting proliferation and invasion of tumor cells. For instance, tumor-derived CXCL5 promoted proliferation and invasion through activation of the ERK1/2, NF-κB and AKT/β-catenin pathways in hepatocellular and prostate cancers^18^, ^25^. However, contradictory results have also been reported in several types of human cancers, such as colorectal cancer. It was reported that elevated CXCL5 was a significant and independent prognostic factor of survival in all colorectal cancer patients and that CXCL5 promoted the proliferation, migration and partial invasion of cancer cells^26^, while in another study, high expression of CXCL5 was significantly associated with a good prognosis of colorectal cancer patients, possibly due to intratumoral CD8^+^ T cell infiltration^27^. The role of CXCL5 in the progression and prognosis of PDAC has not yet been reported. A previous study by Li et al. showed a correlation between CXCL5 expression and prognosis^28^. They showed that the overexpression of CXCL5 was significantly correlated with poorer tumor differentiation and short patient survival. But in their study, CXCL5 staining was scored as the percentage of tumor cells staining positively for CXCL5, while in more studies^11^, ^18^, ^29^, ^30^, the staining score of CXCL5 for each tissue was calculated by multiplying the intensity of immunostaining score and the percentage of immunoreactive cells score, just like we did. In our study, we further investigated the effects of CXCL5 expression on the prognosis of PDAC and the correlation between CXCL5 expression and clinical pathological parameters and immune cell infiltration by analyzing online databases and performing immunohistochemical staining for CXCL5 and immune cell markers. Our study demonstrated that CXCL5 was overexpressed in PDAC tissues and that a high expression level was associated with a poor prognosis. These results were also proven with the TCGA online databases. High CXCL5 expression in tumor tissues was significantly associated with the T3 stage, the N2 stage and a poor differentiation status. The multivariate Cox regression analysis also showed that CXCL5 expression was an independent prognostic marker in the high CA242 subgroup. The combination of CA242 and CXCL5 expression could be a better prognostic index than CA242 alone for PDAC patients, indicating that CXCL5 might be used as an important supplement to CA242 in the diagnosis of PDAC.

CXCL5 has been reported to participate in shaping tumor immune environment by inducing neutrophil migration through interaction with its receptor, CXCR2^31^. In laryngeal squamous cell carcinoma, neutrophils mediated by CXCL5 promoted tumor cells to escape immune surveillance by inhibiting T cell proliferation and cytokine secretion^32^. In melanoma, tumor-derived CXCL5 recruited high amounts of neutrophils and significantly increased lymph node metastases^33^. In cholangiocarcinoma, CXCL5 was identified to act as a factor in the interaction between cholangiocarcinoma and cancer-associated fibroblasts^34^. In our study, we first reported that high CXCL5 expression in tumor tissues was associated with the increased infiltration of M2 polarized macrophages, neutrophils and IgG^+^ plasma cells. M2 polarized macrophages have been demonstrated to play an important role in carcinogenesis^35^, ^36^. It was reported that CXCL5 modulated macrophage activation, enhanced cholesterol efflux activity in macrophages and limited macrophage foam cell formation in atherosclerosis^37^. Tumor-educated B cells could promote breast cancer lymph node metastasis by producing pathogenic IgG^38^. Our data showed the correlation between CXCL5 expression and suppressive immune cells, suggesting its role in shaping tumor immune microenvironment in PDAC. Further study needed to be explored in the impacts and mechanisms of CXCL5 on the TIME in PDAC.

Overall, our findings suggested CXCL5 could be served as a potential novel prognosis marker for PDAC. Meanwhile, we revealed underlying hints for CXCL5's role in shaping immunosuppressive tumor microenvironment in PDAC. Thus, these findings are potentially valuable in advancing not only our current understanding of TIME, but also the translational use in PDAC prognosis.

## Figures and Tables

**Table 1:** Relationship between CXCL5 expression and clinicopathologic features of PDAC

Variables	Number	Tumoral CXCL5 expression		P value
		Low	High	
Age				
≤60 y	43	22	21	0.580
>60 y	47	24	23	
Gender				
Male	57	28	29	0.391
Female	33	18	15	
T stage				
T1-2	75	42	33	0.036*
T3	15	4	11	
N stage				
N0-1	36	24	12	0.014*
N2	54	22	32	
Differentiation				
Well/moderate	66	40	26	0.003*
Poor	24	6	18	
Perineural invasion				
Negative	71	39	32	0.127
Positive	19	7	12	
CA19-9				
<34 U/ml	26	15	11	0.287
≥34 U/ml	64	31	33	
CA242				
<20 U/ml	42	22	20	0.494
≥20 U/ml	48	24	24	

**Table 2:** Univariate and multivariate analyses of CXCL5 expression and clinicopathological parameters

Variables	Univariate analysis			Multivariate analysis		
	HR	95%CI	P value	HR	95%CI	P value
CXCL5 expression						
High vs Low	2.366	1.405-3.983	0.001*	1.565	0.889-2.754	0.120
Age (years)						
≤60 vs >60	0.683	0.411-1.135	0.141			
Gender						
Male vs Female	1.017	0.601-1.720	0.951			
T stage						
T3 vs T1-2	2.524	1.342-4.747	0.004*	2.536	1.286-5.001	0.007*
N stage						
N2 vs N0-1	2.69	1.548-4.674	0.000*	2.144	1.179-3.898	0.012*
Differentiation						
Poor vs Well/moderate	2.518	1.472-4.307	0.001*	2.342	1.312-4.182	0.004*
Perineural invasion						
Positive va Negative	1.373	0.740-2.548	0.315			
CA19-9 (U/ml)						
≥34 vs <34	3.282	1.611-6.684	0.001*	2.495	1.092-5.703	0.030*
CA242 (U/ml)						
≥20 vs <20	2.056	1.204-3.510	0.008*	1.304	0.706-2.409	0.397

## Abbreviations

CHOL: cholangiocarcinoma
COAD: colon adenocarcinoma
CXCL5: C-X-C motif chemokine 5
CXCR2: C-X-C chemokine receptor type 2
DMEM: Dulbecco's modified Eagle's medium
ESCA: esophageal carcinoma
FBS: Fetal bovine serum
GEO: Gene Expression Omnibus
GEPIA: Gene Expression Profiling Interactive Analysis
GTEx: Genotype-Tissue Expression
HFP: High-power field
IGHG1: Immunoglobulin heavy constant gamma 1
OS: Overall survival
PAAD: Pancreatic adenocarcinoma
PDAC: Pancreatic ductal adenocarcinoma
READ: Rectum adenocarcinoma
ROC: Receiver operating characteristic
STAD: Stomach adenocarcinoma
TAMs: Tumor-associated macrophages
TANs: Tumor-associated neutrophils
TCGA: The Cancer Genome Atlas
TIME: Tumor immune microenvironment
Tregs: Regulatory T cells
